# Topical Meglumine Antimoniate Gel for Cutaneous Leishmaniasis: Formulation, Evaluation, and In Silico Insights

**DOI:** 10.3390/gels11080601

**Published:** 2025-08-01

**Authors:** Lilian Sosa, Lupe Carolina Espinoza, Alba Pujol, José Correa-Basurto, David Méndez-Luna, Paulo Sarango-Granda, Diana Berenguer, Cristina Riera, Beatriz Clares-Naveros, Ana Cristina Calpena, Rafel Prohens, Marcelle Silva-Abreu

**Affiliations:** 1 Instituto de Investigaciones en Microbiología (IIM), Facultad de Ciencias, Universidad Nacional Autónoma de Honduras (UNAH), Tegucigalpa 11101, Honduras; liliansosa2012@gmail.com; 2 Facultad de Ciencias Químicas y Farmacia, Universidad Nacional Autónoma de Honduras (UNAH), Tegucigalpa 11101, Honduras; 3 Departamento de Química, Facultad de Ciencias Exactas y Naturales, Universidad Técnica Particular de Loja, San Cayetano Alto, Loja 1101608, Ecuador; lcespinoza@utpl.edu.ec (L.C.E.); pcsarango@utpl.edu.ec (P.S.-G.); 4 Institut de Nanociència i Nanotecnologia, Universitat de Barcelona (UB), Av. Diagonal 645, 27-31, 08028 Barcelona, Spain; beatrizclares@ugr.es (B.C.-N.); anacalpena@ub.edu (A.C.C.); 5 Laboratory of Parasitology, Department of Biology, Health, and Environment, Faculty of Pharmacy and Food Sciences, University of Barcelona (UB), Av. Diagonal 645, 27-31, 08028 Barcelona, Spain; apujolbrugues@gmail.com (A.P.); berenguer.diana@gmail.com (D.B.); mcriera@ub.edu (C.R.); 6 Laboratorio de Diseño de Nuevos Fármacos e Innovación Biotecnológica, Escuela Superior de Medicina, Instituto Politécnico Nacional, Plan de San Luis Díaz Mirón, Ciudad de México 11340, Mexico; corrjose@gmail.com; 7 Departamento de Fisiología, Escuela Nacional de Ciencias Biológicas, Instituto Politécnico Nacional, Zacatenango, Av. Wilfrido Massieu 399, Col. Nueva Industrial Vallejo, Alcaldía Gustavo A. Madero, Ciudad de México 07738, Mexico; meld8909@gmail.com; 8 Department of Pharmacy and Pharmaceutical Technology, School of Pharmacy, University of Granada, 18071 Granada, Spain; 9 Departament de Farmàcia i Tecnologia Farmacèutica, i Fisicoquímica, Facultat de Farmàcia i Ciències de l’Alimentació, Universitat de Barcelona (UB), Av. Diagonal 645, 27-31, 08028 Barcelona, Spain; 10 Laboratory of Organic Chemistry, Faculty of Pharmacy and Food Sciences, University of Barcelona, Avda, Joan XXIII, 08028 Barcelona, Spain; rafel_prohens@ub.edu

**Keywords:** meglumine antimoniate, skin, Gels, Pluronic^®^ F127, physicochemical characterization, Leishmanicidal activity, *Leishmania infantum*

## Abstract

Leishmaniasis is an infectious disease common in tropical and subtropical regions worldwide. This study aimed to develop a topical meglumine antimoniate gel (MA-gel) for the treatment of cutaneous leishmaniasis. The MA-gel was characterized in terms of morphology, pH, swelling, porosity, rheology, and thermal properties by differential scanning calorimetry (DSC). Biopharmaceutical evaluation included in vitro drug release and ex vivo skin permeation. Safety was evaluated through biomechanical skin property measurements and cytotoxicity in HaCaT and RAW 267 cells. Leishmanicidal activity was tested against promastigotes and amastigotes of *Leishmania infantum*, and in silico studies were conducted to explore possible mechanisms of action. The composition of the MA-gel included 30% MA, 20% Pluronic^®^ F127 (P407), and 50% water. Scanning electron microscopy revealed a sponge-like and porous internal structure of the MA-gel. This formula exhibited a pH of 5.45, swelling at approximately 12 min, and a porosity of 85.07%. The DSC showed that there was no incompatibility between MA and P407. Drug release followed a first-order kinetic profile, with 22.11 µg/g/cm^2^ of the drug retained in the skin and no permeation into the receptor compartment. The MA-gel showed no microbial growth, no cytotoxicity in keratinocytes, and no skin damage. The IC_50_ for promastigotes and amastigotes of *L. infantum* were 3.56 and 23.11 µg/mL, respectively. In silico studies suggested that MA could act on three potential therapeutic targets according to its binding mode. The MA-gel demonstrated promising physicochemical, safety, and antiparasitic properties, supporting its potential as a topical treatment for cutaneous leishmaniasis.

## 1. Introduction

Leishmaniasis is a parasitic disease of significant public health relevance worldwide. It is caused by protozoa of the Leishmania genus and is transmitted through the bite of infected sandflies [1]. This disease has a broad geographical distribution, primarily affecting tropical and subtropical regions, with high incidence rates in Latin American countries [2]. According to estimates from the World Health Organization (WHO), between 600,000 and 1,000,000 new cases of leishmaniasis are reported annually, resulting in substantial morbidity and a considerable socioeconomic burden in affected communities [3]. In its biological cycle, Leishmania exhibits two evolutionary forms: promastigotes and amastigotes [4,5].

Cutaneous leishmaniasis is characterized by the development of ulcerative skin lesions, which may become chronic and leave permanent scars, affecting the patients’ physical, psychological, and social well-being [6,7]. There are several clinical forms of the disease: localized cutaneous leishmaniasis (Oriental button), which begins as a small erythematous papule at the bite site that enlarges, ulcerates centrally, and presents a raised, hyperpigmented border; relapsing leishmaniasis, a rare form associated with specific *Leishmania* species; diffuse cutaneous leishmaniasis, a disseminated and chronic infection characterized by non-ulcerative plaques, papules, and nodules primarily on the face and extremities; and mucosal leishmaniasis (espundia), which initially affects the nasal mucosa and, if untreated, can cause progressive destruction of the nasal septum, palate, lips, pharynx, and larynx [8].

**Figure 1:**
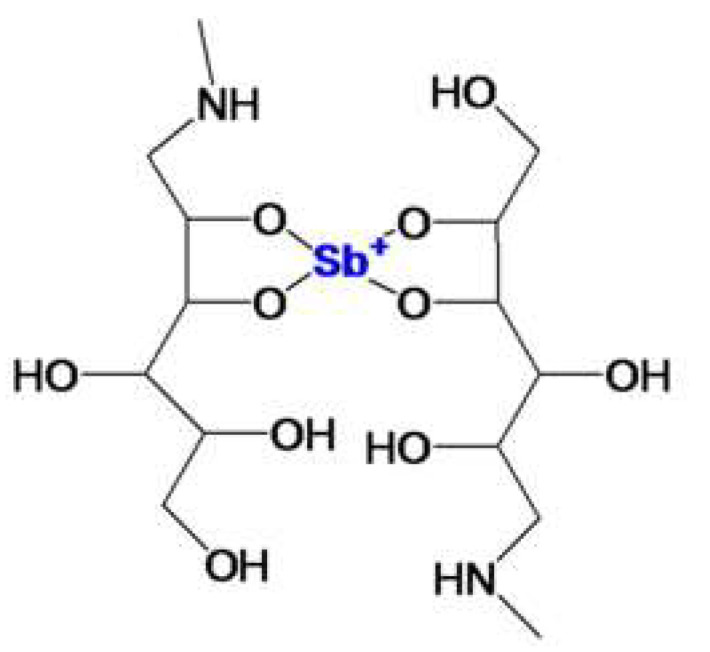
Meglumine antimoniate chemical structure.

The treatment of this disease depends on several factors, including the Leishmania species involved, the location and severity of the lesions, and the patient’s immune response [9,10]. Pentavalent antimonials, such as meglumine antimoniate (MA) (Figure 1), have been used for decades as the first-line therapeutic option [11,12]. However, systemic administration (intravenous or intramuscular) presents limitations due to serious adverse effects, including liver disorders, nephrotoxicity, pancreatitis, myalgia and arthralgia, cardiovascular toxicity, and local reactions at the injection site [13]. MA has a molecular weight of 365.98 g/mol and is a class III drug (high solubility and low permeability) according to the biopharmaceutical classification system [14]. It is not absorbed orally but shows >90% bioavailability via intramuscular and subcutaneous routes. Regarding the resorption of this drug, it exhibits a biphasic elimination pattern with a terminal half-life of 76 h, likely due to its hepatic conversion into trivalent antimony, which accumulates in specific tissues and is slowly released [15]. Treatment adherence may be compromised due to the pain and discomfort associated with parenteral administration [16]. Despite its proven efficacy, MA treatment is usually restricted to hospitalized patients under medical supervision [17]. Considering these limitations, the development of topical formulations based on this molecule represents a promising alternative to improving treatment efficacy and safety.

The use of topical MA administration would offer benefits such as localized administration directly to the cutaneous leishmaniasis skin lesion, thereby minimizing systemic side effects of this drug and improving bioavailability at the site of action. It also prevents first-pass liver damage, maintains a constant release over time, and has a lower rate of treatment discontinuation due to ease of application. The topical form would be applied only in cases of cutaneous leishmaniasis and not in visceral leishmaniasis, where systemic treatments are necessary [18]. Current topical formulations of MA reported in the scientific literature are limited, highlighting the need to explore innovative delivery systems that enhance drug stability and skin permeability. Over the past decade, key studies have utilized gel-based Sepigel^®^ formulations, polymeric colloidal nanocarriers composed of maltodextrin, and nanoparticles [19,20,21].

Pluronic^®^ F127 (P407) has been shown to enhance the incorporation of drugs into poloxamer matrices, thereby improving bioavailability, reducing toxicity, and increasing therapeutic efficacy [13]. This poloxamer exhibits thermoreversible behavior, forming liquid solutions at low temperatures and semisolid gels at physiological temperatures, facilitating application and skin retention. Additionally, it shows excellent compatibility with both hydrophobic and hydrophilic active pharmaceutical ingredients, enabling the stabilization of a diverse range of drug types. These characteristics make it an ideal candidate for formulating controlled-release delivery systems. Moreover, its application on healthy and damaged skin has shown promising results in administering antimicrobial and antiparasitic agents [22].

This study aims to develop and physicochemically characterize a meglumine antimoniate gel (MA-gel) formulated with P407, evaluate its biopharmaceutical properties, assess its cytotoxicity and skin tolerability, and confirm its leishmanicidal activity against promastigote and amastigote forms of *L. infantum*, suggesting some biological targets according to in silico simulations, thereby proposing a potential therapeutic alternative for the treatment of severe cutaneous leishmaniasis.

## 2. Results and Discussion

### 2.1. Preparation of MA-Gel and Gelation Time

**Figure 2:**
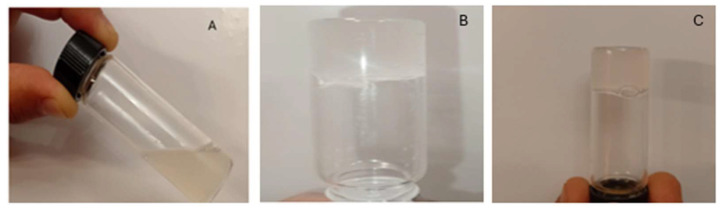
Physical appearance of MA-gel. (**A**) 4 °C. (**B**) 25 °C. (**C**) 32 °C.

The resulting gel was whitish and translucent, liquid at 4 °C, gelatinous at 25 °C, and firm at 32 °C (Figure 2). The gelation time was approximately 30 s, enabling a controlled transition from the liquid to the gel phase upon contact with the skin, neither immediate, which would hinder application, nor excessively slow, which could lead to runoff. The self-assembly and gelation of P407 in aqueous solutions occur through an endothermic transition, primarily driven by entropy increases associated with the dehydration of hydrophobic blocks and micelle formation [23]. No differences in gelation time were observed between the formulation containing MA (MA-gel) and the control without MA (P407-gel).

### 2.2. Physicochemical Characterization of MA-Gel

#### 2.2.1. Morphological Analysis

**Figure 3:**
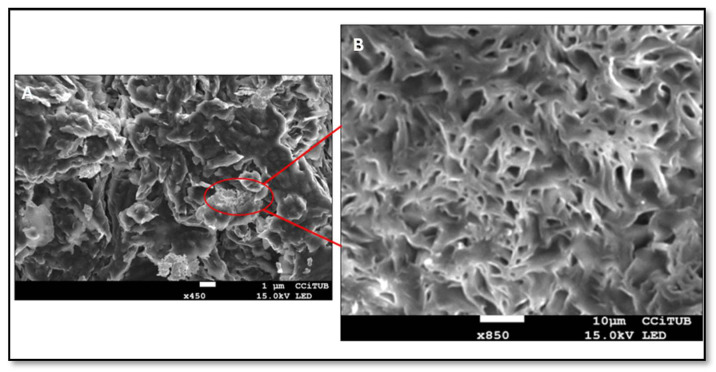
Scanning electron microscopy of MA-gel. (**A**) 450×, scale bar 1 µm, (**B**) 850×, scale bar 10 µm.

Scanning electron microscopy revealed that the internal macroscopic structure of MA-gel resembled a sponge-like architecture, which is characteristic of P407-based gels (Figure 3A,B) [24,25,26]. This morphology is consistent with the three-dimensional structure or networks typical of polymeric gels, where the arrangement of polymer chains determines the size and shape of the network, thus defining the porosity of the matrix [27]. This approach is important because the porosity of gels influences properties such as swelling, drug release kinetics, and overall performance of the gel [28].

#### 2.2.2. pH Values

The pH value was suitable for skin application, with no significant differences observed between values ranging from 5.40 ± 0.09 to 5.45 ± 0.12 (*p* > 0.05) at 4 °C and those from 5.43 ± 0.11 to 5.47 ± 0.19 (*p* > 0.05) at 32 °C. Moreover, these values remained constant after three months of storage.

#### 2.2.3. Swelling and Degradation Tests

**Figure 4:**
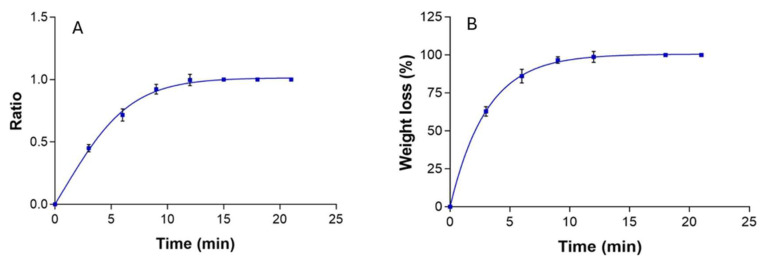
Swelling behavior and degradation kinetics of MA-gel. (**A**) Swelling ratio. (**B**) Degradation profile.

The swelling behavior of the MA-gel followed a sigmoidal Boltzmann kinetic model, with a kinetic constant k = 0.97 min^−1^ (r^2^ = 0.98) (Figure 4A). The degradation process was completed within 12 min and fitted a first-order model with a kinetic constant of 0.32 min^−1^ (r^2^ = 0.99) (Figure 4B). Compared to previous studies utilizing P407 as a drug delivery vehicle, the MA-gel exhibited a faster swelling rate [25,29]. In this study, complete swelling of the MA-gel was achieved within 12 min. Additionally, the porosity percentage of the MA-gel was ~85.07 ± 0.43%. Furthermore, the porosity of the MA-gel was approximately 85.07 ± 0.43%, consistent with the porous structure observed in the SEM images.

### 2.3. Rheological Characterization: Rotational and Oscillatory Tests

#### 2.3.1. Rotational Test

**Figure 5:**
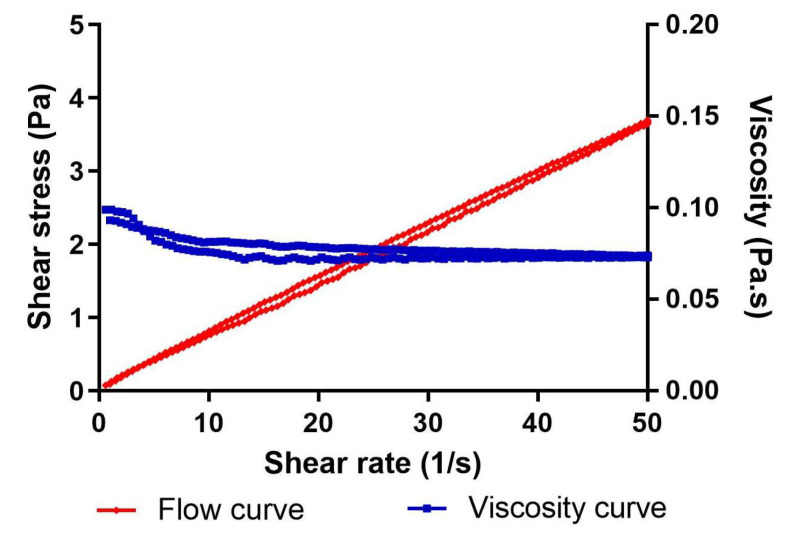
Rheological behavior of MA-gel analyzed under rotational conditions at 4 °C.

At 4 °C, the MA-gel exhibited shear-thinning behavior characteristic of pseudoplastic materials. Viscosity progressively decreased with an increasing shear rate, and no significant thixotropic behavior was observed. The Cross model provided the best fit for the flow data, with high correlation coefficients (r^2^ > 0.99) and a viscosity value of 78.81 ± 0.09 mPa.S (Figure 5). At 25 and 32 °C, the formulation transitioned to a semi-solid gel state, resulting in increased consistency and adhesiveness, which prevented homogeneous flow within the cone–plate gap. These findings are consistent with previous studies using P407, particularly at low storage temperatures (4 °C), where the formulation remains more fluid and easily spreadable [29,30].

#### 2.3.2. Oscillatory Test

**Figure 6:**
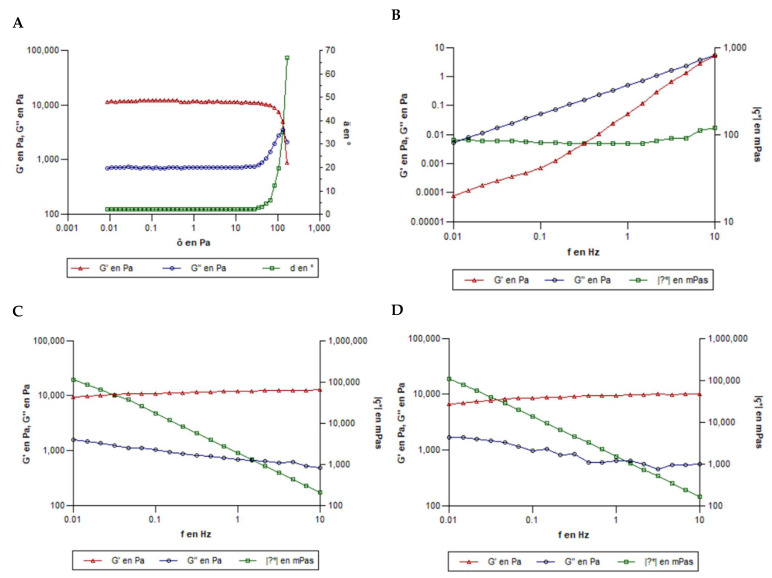
Oscillatory tests. (**A**) Breakdown in the MA-gel structure. (**B**) Oscillatory measurements applied to the MA-gel at 4 °C. (**C**) Oscillatory measurements were applied to the MA-gel at 25 °C. (**D**) Oscillatory measurements applied to the MA-gel at 32 °C. G′: storage modulus, G″: loss modulus, and η*: complex viscosity.

The temperature sweep performed using oscillatory rheological testing confirmed the temperature-sensitive behavior of MA-gel and revealed that the formulation begins to gel at 19 °C and completes gelation around 25 °C. From the amplitude sweep tests at 25 °C and 32 °C, the linear viscoelastic region (LVR) is defined as the range in which G′ and G″ remain constant and the phase angle (δ) is stable. The curves obtained at both temperatures are nearly superimposable, indicating similar viscoelastic behavior; however, the LVR extends further at 32 °C, up to approximately 100 Pa, compared to 40 Pa at 25 °C. Within these limits, the gel maintains its internal structure (G′ > G″, low δ). Beyond the critical stress values, G′ decreases, G″ increases, and δ rises sharply, indicating the onset of structural breakdown (Figure 6A). These findings confirm that the formulation exhibits strong gel-like behavior and good mechanical stability, with enhanced structural resistance at higher temperatures.

Frequency sweep tests at 4 °C (Figure 6B) showed G″ > G′ across most of the frequency range, indicating a predominantly viscous behavior. Both G′ and G″ increased with frequency, and at 10 Hz, the two moduli approached each other, suggesting an incipient gel-like structure at high frequencies. At 25 °C and 32 °C (Figure 6C,D), the behavior reversed, with G′ > G″ throughout the frequency range and G′ remaining nearly constant, indicating the formation of a structured, elastic network characterized by an elastic plateau.

These findings confirm the thermoreversible gelation properties of P407, which forms micellar networks at physiological temperatures due to increased hydrophobic interactions among PPO blocks [31]. At 4 °C, the formulation displayed frequency-dependent viscous behavior, whereas at 32 °C, it exhibited frequency-independent elastic behavior consistent with a physically crosslinked gel structure. These rheological characteristics suggest that the MA-gel could be used as a sprayable, thermoresponsive topical system that undergoes a sol–gel transition to form a stable gel at body temperature [32].

### 2.4. Extensibility Test

**Figure 7:**
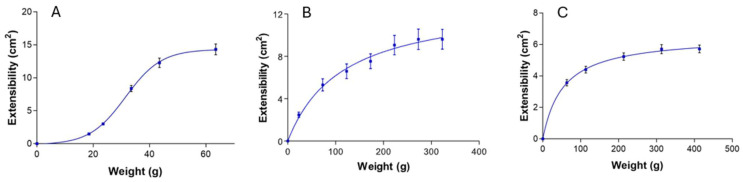
Extensibility studies. (**A**) MA-gel at 4 °C. (**B**) MA-gel at 25 °C. (**C**) MA-gel at 32 °C.

Figure 7 presents the extensibility profiles of the MA-gel measured at 4, 25, and 32 °C, showing a sigmoidal Boltzmann model (4 °C) and a hyperbolic model (25 and 32 °C). At 4 °C, the gel exhibited significantly greater extensibility under lower applied weights compared to its behavior at 25 and 32 °C. This enhanced extensibility is attributed to the lower viscosity of the formulation at low temperatures. Such behavior is consistent with previous studies and is characteristic of P407-based systems. This thermoresponsive property supports the potential application of MA-gel in spray format, enabling administration without direct contact with Leishmania-infected lesions. Additionally, the gel’s cooling effect may offer soothing benefits when applied to ulcerated skin, which is often associated with cutaneous leishmaniasis.

### 2.5. Thermal Characterization and Compatibility Studies of MA-Gel by Differential Scanning Calorimetry (DSC)

**Figure 8:**
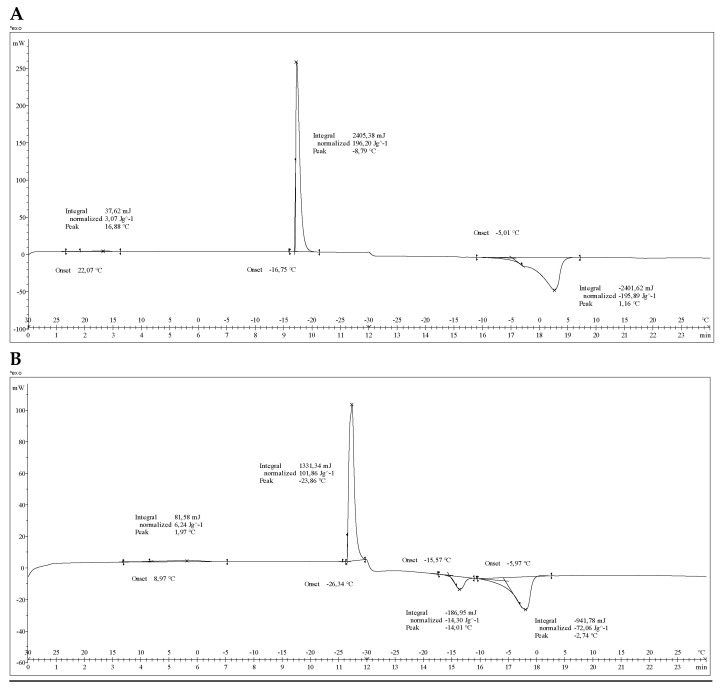
Thermal characterization. (**A**) DSC thermogram of P407-gel. (**B**) DSC thermogram of MA-gel.

MA-gel and blank gel (P407-gel) exhibited a common exothermic phenomenon upon cooling to −30 °C, which could be attributed to the solidification of the gel matrix. The presence of MA in the gel shifted this event to a lower temperature: while the P407 blank gel showed an exothermic peak at −16.8 °C, the MA-gel exhibited this peak at −26.3 °C. Upon reheating to 30 °C, P407-gel displayed a broad endothermic event starting at −5 °C, attributed to overlapping melting and gelation phenomena. In contrast, the MA-gel presented two well-defined endothermic events at −15.6 °C and −6.0 °C, suggesting more complex thermal behavior due to the presence of MA within the gel network (Figure 8A,B).

**Figure 9:**
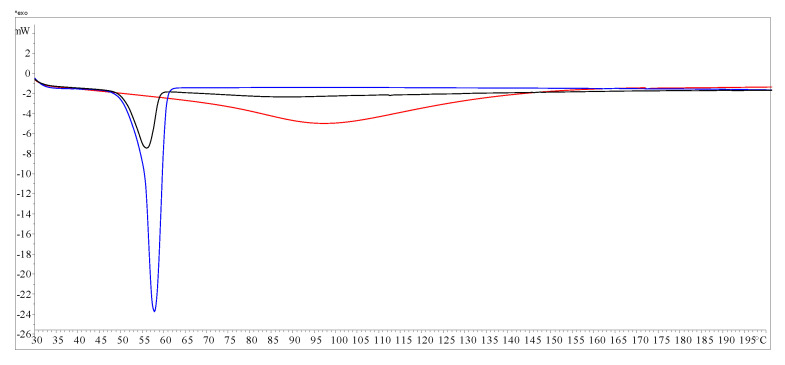
DSC thermograms obtained in the study of potential meglumine antimoniate (MA)/Pluronic^®^ F127 (P407) interactions. MA (red); P407 (blue); mixture of the components of the formulation in the original proportion (black).

To further investigate the potential compatibility between MA and P407, DSC thermograms of the pure components and their mixture (in the same proportion used for gel preparation) were recorded (Figure 9). DSC is frequently used in the study of potential drug–excipient interactions [33,34]. In general, the assessment is based on modifications in the thermal behavior of mixtures compared to pure components. DSC criteria for the interaction/compatibility between an active ingredient and an excipient establishes that there is no interaction; therefore, the species are considered compatible when the thermogram of the mixture is a superposition of the individual thermograms, whereas when a new thermal phenomenon appears at a lower temperature than the first thermal phenomenon observed for any of the species separately, it is accepted that there is an interaction, and the species are considered incompatible [35,36].

The DSC thermogram of P407 showed a well-defined endotherm starting at 50.2 °C with an associated heat of 120.7 J/g, corresponding to its melting process. On the other hand, MA exhibited a broad endotherm starting at 54.6 °C with an associated heat of 266.7 J/g (see Supplementary Information, Figures S1–S3). The DSC thermogram of the mixture showed an almost perfect superposition of the thermal events of the individual components, indicating no significant thermal interaction under the tested conditions. These results suggest that MA and P407 are thermally compatible in this formulation, and thus, the stability and performance of the MA-gel are not compromised by incompatibility between the drug and the excipient.

### 2.6. In Vitro Release Studies

**Figure 10:**
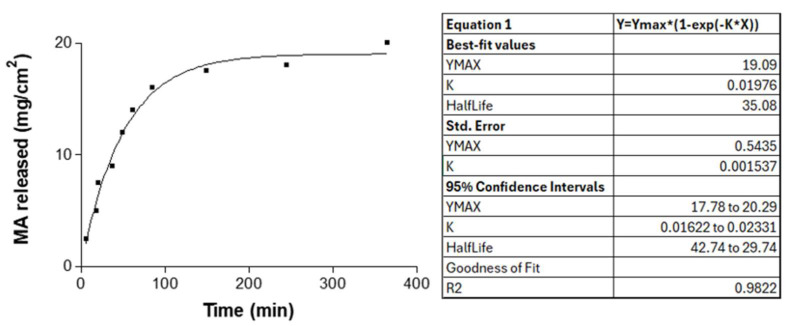
The in vitro release profile from the MA-gel.

The release profile of MA from the gel was slightly faster in the first 100 min, then it became steady over the next 300 min. After 6 h, 20 mg/cm^2^ of the active ingredient was released, following a first-order mathematical model (Fickian kinetics) with a maximum release amount (Ymax) of 19.04 mg/cm^2^ and a release constant (K) of 0.02 h^−1^ (Figure 10). The release rate of MA in this study was greater than that reported in a previous study, which involved a lower quantity of drug released over a more extended period (55 h) [19]. This result could be attributed to the use of P407, which generates more porous systems than other polymers. The first-order release kinetic profile of MA from P407 gel follows a concentration gradient pattern, in which the drug release rate through a membrane is directly proportional to the amount of drug remaining in the diffusion area [19].

### 2.7. Ex Vivo Permeation Studies

The ex vivo permeation studies showed that the MA did not permeate through the skin but remained retained within it. This could be due to the liposolubility of the epidermis, which probably caused the antimony to take time to pass through this layer of the skin, as it is a more hydrophilic molecule. Some studies have shown small amounts of permeation in undamaged human skin [19]. Other studies have revealed that a small amount of pentavalent antimony (Sb^V^) permeates into the receptor compartment, and a high percentage is retained in ex vivo permeation studies using rat skin, which is more permeable than human skin [37,38]. Regarding the amount of drug retained, the results showed that 22.11 µg/g/cm^2^ of MA was retained within the skin after 27 h of assay. This result indicates that the drug was able to cross the stratum corneum and remain retained within it, forming a reservoir that allows for a local effect and a slow release of the drug into the dermis, where the parasite is located. Moreover, the excipient P407 has a depot effect, promoting the local activity of the drug retained within the skin [25].

### 2.8. Microbiological Quality Control

**Figure 11:**
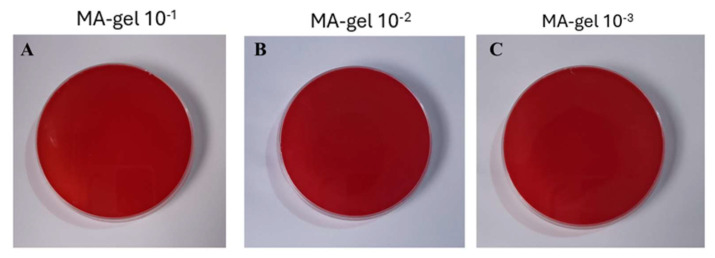
Blood agar plates were seeded at three different concentrations, showing no bacterial growth. (**A**) MA-gel concentration 10^−1^. (**B**) Gel concentration at 10^−2^. (**C**) MA-gel concentration 10^−3^.

Microbiological control is a crucial test to determine whether the formulation is suitable for administration in a specific area. In this particular case, it is necessary to ensure the microbiological quality of the gel because it would be applied to an area infected with leishmaniasis. Therefore, a formulation contaminated by fungi or bacteria could lead to superinfection at the application site. The results of this assay showed that there was no bacterial or fungal growth in the different culture media used. *Staphylococcus aureus* and *Pseudomonas aeruginosa* were absent, as established by the United States Pharmacopeia. Figure 11 shows three blood agar plates at concentrations of 10^−1^, 10^−2^, and 10^−3^ with no bacterial growth after 48 h of incubation.

### 2.9. In Vivo Tolerance Study

**Figure 12:**
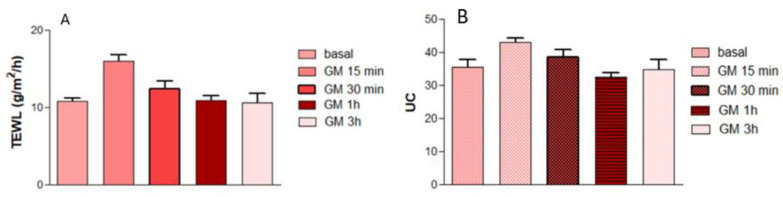
The evolution of biomechanical parameters is monitored before application (baseline) and immediately after P407 gel application. (**A**) TEWL values in g/m^2^/h. (**B**) SCH values in corneometric units UC.

The results of the skin biomechanical parameters evaluated are shown in Figure 12. Regarding TEWL, an increase in this parameter was observed 15 min after gel application with respect to the basal state. This is because the gel had not entirely dried after application to the skin, resulting in values similar to those observed when transepithelial water loss occurs. However, this parameter decreased significantly after 30 min, returning to basal values after 3 h, indicating that the gel does not alter the stratum corneum. On the other hand, when measuring the hydration of the stratum corneum (SCH), an increase in corneometric units is observed 15 and 30 min after gel application compared to basal values, since the gel was still wet. At the first hour, a decrease in the values compared to the basal value was observed. This is because the gel forms a film upon complete drying. However, as time passes, the values become more similar to the basal value. No itching, burning, or redness was observed when P407 gel was applied, indicating that this product is suitable for skin application. P407 is known to be widely used as a drug vehicle applied to the skin without causing damage [39,40,41,42]. It has been shown to promote fibroblast accumulation, granulation tissue formation, and angiogenesis, which would be beneficial in cases of ulcerative leishmaniasis [43]. Additionally, it is considered biodegradable and biocompatible [44].

### 2.10. Cytotoxicity Studies

**Figure 13:**
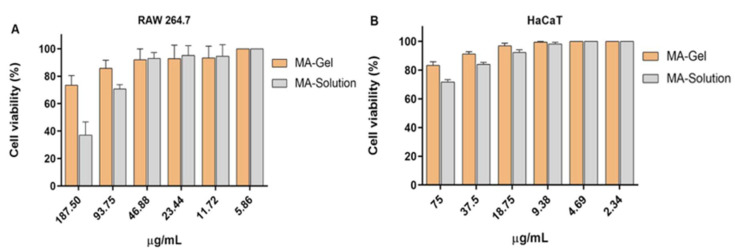
Cytotoxicity studies in two cell lines. (**A**) RAW 264.7 macrophages. (**B**) HaCaT keratinocytes.

Figure 13A,B illustrate the cytotoxic effects on the analyzed cell lines using the MA-gel and MA-solution. Specifically, no cytotoxicity was monitored in the HaCaT keratinocyte cell line at the dilution ranges analyzed for the MA-gel. Cytotoxicity was only observed with the MA-solution at a concentration of 75 µg/mL. On the other hand, the MA-gel and MA-solution did not exhibit cytotoxicity in the RAW264.7 cell line at concentrations up to 93.75 µg/mL and 46.88 µg/mL, respectively. These results are similar to those observed in previous studies [19]. The toxicity of MA-gel could be attributed to the presence of P-glycoprotein in RAW 264.7 cells, also known as MDR1 or ABCB1, a protein located in cell membranes that facilitates the transport of various substances across them. It is particularly known for its role in multidrug resistance; this behavior has also been seen in studies where P207 has been used as a gel vehicle [45].

### 2.11. Leishmanicidal Activity in Both Stages of L. infantum Parasite

The IC50 value found for the MA-solution against *L. infantum* promastigotes was 6.96 ± 15.98 µg/mL, a higher value than that found for the MA-gel, which was 3.56 ± 0.22 µg/mL. The same behavior was observed with *L. infantum* amastigotes, where the IC50 was lower for the MA-gel than for the MA-solution (Table 1). Regarding the selectivity index (SI) values, the higher the SI value, the greater the leishmanicidal activity. Table 1 shows that the MA-gel is more effective against *L. infantum* than the MA-solution. The IC_50_ values in *L. infantum* promastigotes are lower than those found in a previous study with emulgels [19]. This finding could be attributed to the use of P407, which is more porous than emulgels, resulting in a faster release and, consequently, a faster effect. The IC_50_ values found were higher than those reported in the study regarding amastigotes. This could be explained by the type of cells used for the promastigote infection, RAW 264.7, which are more resistant to drugs; therefore, the IC_50_ values are higher than those obtained when other cell types are used. Finally, the IC_50_ values against amastigotes found in this study were similar to those reported previously [38].

### 2.12. Computational Studies

**Figure 14:**
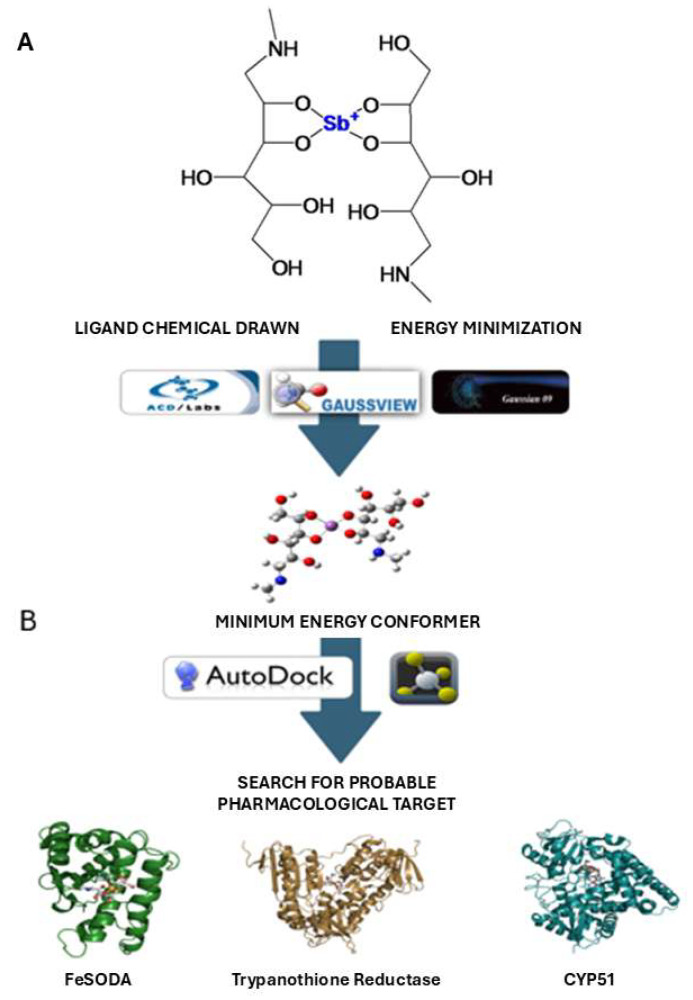
Workflow of the in silico simulations. (**A**) MA chemical structure. (**B**) Probable targets as hydrogen bond acceptors analyzed through molecular docking simulations.

Understanding the atomistic interactions between ligands and proteins is essential for elucidating the molecular and pharmacological mechanisms of drug action. Using molecular docking simulations, we investigated the probable pharmacological targets of MA based on its chemical structure following a known docking protocol that includes ligand and protein preparation to perform docking simulations (Figure 14).

**Figure 15:**
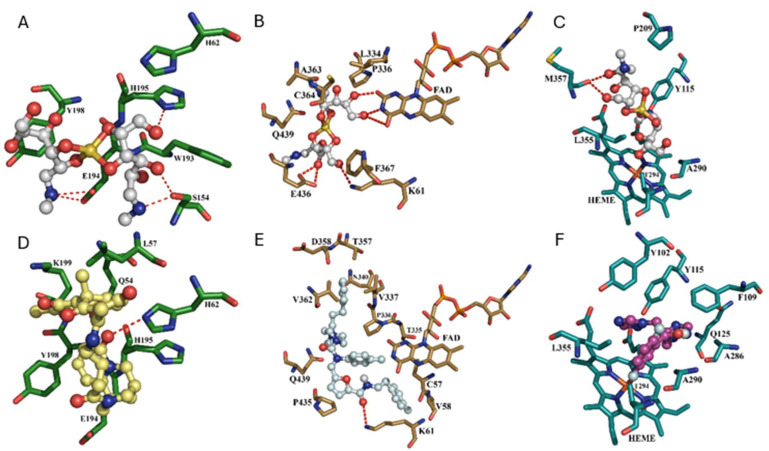
Binding modes of MA and some known inhibitors on the probable pharmacological targets. (**A**) On FeSODA. (**B**) On Trypanothione reductase. (**C**) On CYP51. (**D**) ZINC000253403245 on FeSODA. (**E**) C9 on Trypanothione reductase. (**F**) Fluconazole on CYP51. H-bonds are depicted as red dashes.

For FeSODA, MA binds near residues that comprise the active site of *L. major* FeSODA (PDB ID: 4F2N), including H62, S154, W193, E194, and H195. Our results show hydrogen bond interactions with side chains of S154, E194, H195, and Y198, highlighting the relevance of these residues in ligand recognition (Figure 15A). The ligand recognition properties are governed as hydrogen bond donators due to hydroxyl groups that occur with other biological targets such as trypanothione reductase and CYP51, which contain chemical groups working as hydrogen bond acceptors (Figure 15B,C). Notably, this binding mode is highly similar to that of ZINC000253403245, a compound identified through advanced computational drug discovery methods [8], which also exhibits pivotal interactions with residues such as H62 (via hydrogen bonding), E194, H195, and Y198 (collectively forming the binding site on the FeSODA) (Figure 15D). In the case of trypanothione reductase (PDB ID: 2JK6), MA forms hydrogen bonds with K61 and E436 (by forming two H-bonds), as well as exhibiting three hydrogen bond interactions with the FAD cofactor, essential for enzymatic activity [46]. These interactions suggest a possible mechanism by which MA may inhibit trypanothione reductase function, disrupting its catalytic cycle (Figure 15B). This premise gains significance when compared to the binding mode of C9 (4-(((5-((4-Fluorophenethyl)carbamoyl)furan-2-yl)methyl)(4-fluorophenyl)carbamoyl)-1-methyl-1-(3-phenylpropyl)piperazin-1-ium iodide, which also highlights the importance of reaching the FAD cofactor to disrupt enzyme catalysis. C9 establishes contacts with residues that also appear in the binding mode of MA, such as K61, T335, P336, P435, and Q439, underscoring MA’s significant capability to access the TR enzyme’s binding site (Figure 15E) [47]. Regarding CYP51 (a cytochrome P450 enzyme that catalyzes demethylation at the 14α position of sterol intermediates, a key step in ergosterol biosynthesis), MA exhibits notable binding affinity by making several hydrogen bonds (Figure 15C) [48]. It occupies a binding pocket similar to that of fluconazole (a co-crystallized azole in PDB ID: 3L4D) [49]. Specifically, MA interacts with the heme group and residues Y115, A290, L355, and M357 (by forming two H-bonds), indicating its potential as a CYP51 ligand (Figure 15C). This is further corroborated by the formation of a fluconazole/CYP51 complex under docking studies, which highlights interactions with the heme group and residues A290 and L355, pivotal for CYP51’s molecular recognition of its cognate ligands (Figure 15F).

For all the MA and inhibitor/target complexes analyzed through docking simulations, the interacting residues are presented in Table 2. Lastly, binding energy analysis from docking simulations denotes trypanothione reductase as the most promising pharmacological target for MA, with the following ranking based on binding energy: trypanothione reductase (−5.03 kcal/mol) > CYP51 (−4.58 kcal/mol) > FeSODA (−3.9 kcal/mol). In this context, it is worth mentioning that although the energetic values obtained for MA in all the probable pharmacological targets are lower than those observed for the potential inhibitors of these proteins, this may be due to the chemical nature of MA, which might function as a prodrug requiring catalysis mediated by these targets, followed by the molecular mechanism initially proposed (reduction of Sb(V) to Sb(III)). These results warrant further experimental validation, such as isolated enzyme inhibition assays, to confirm the proposed mechanism.

**Figure 16:**
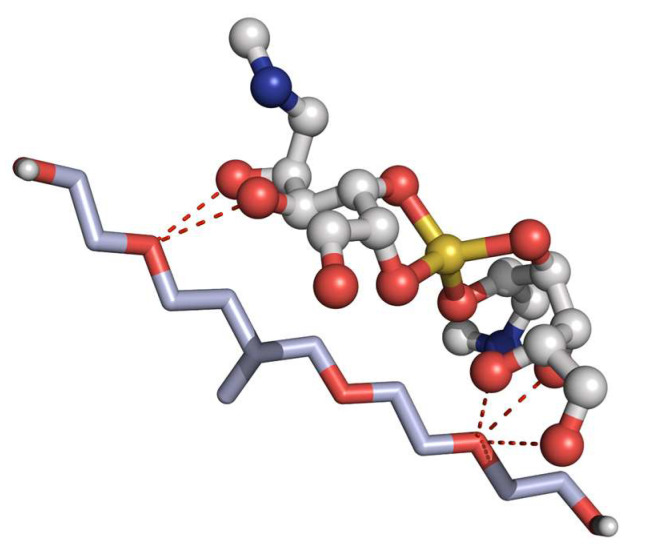
Binding mode of MA for the Pluronic^®^ F-127 (P407) system. H-bonds are depicted as red dashes.

Lastly, analysis of the drug delivery system formed between P407 and MA provides insight into the chemical interactions that govern molecular recognition in this pharmaceutical delivery system. Under docking simulations, the primary interactions observed are hydrogen bonds formed between the PEO blocks of P407 and the oxygen atoms not coordinated to the Sb^+^ atom of MA (Figure 16). These weak interactions appear to be the most significant contributors to the stabilization of the drug delivery system, with an energetic value of −0.29 kcal/mol. However, althought this binding mode is only with a mononer of F-127, it is well-know that F-127 is constituted by several mononers that can accomodate more than one MA by non-bonding interactions.

## 3. Conclusions

This study presents the development of a thermoresponsive meglumine antimoniate gel (MA-gel) using Poloxamer P407 as a promising topical alternative for the treatment of cutaneous leishmaniasis. Unlike conventional systemic therapies associated with high toxicity and poor patient compliance, this formulation enables localized, non-invasive delivery, which can be administered by cold spray, thereby avoiding product contamination and ensuring patient compliance. The MA-gel exhibited rapid gelation, high porosity, and sprayable viscosity, which are novel features that facilitate easy application on ulcerated or sensitive lesions. Notably, the gel achieved high retention of the drug within the skin without systemic diffusion, ensuring targeted action where the parasite resides. MA-gel exhibited no cytotoxicity in keratinocytes, no skin irritation, and significant leishmanicidal activity against both the promastigote and amastigote stages of *L. infantum*. Furthermore, the integration of in silico docking studies provided new possible mechanistic insights, identifying likely molecular targets of MA in *L. infantum*. This innovative gel-based platform not only addresses key limitations of conventional systemic treatments but also contributes to the advancement of safe, effective, and patient-friendly topical therapies for neglected tropical diseases.

## 4. Materials and Methods

### 4.1. Materials

Meglumine antimoniate (MA) was obtained from Acros Organics (Thermo Fisher Scientific, Waltham, MA, USA). Pluronic^®^ F-127 (P407) was obtained from Fagron (Barcelona, Spain). The distilled water used in the experiments was obtained from a Milli-Q Plus system (Millipore Co., Burlington, MA, USA). All the reagents were of analytical grade.

### 4.2. Preparation of MA-Gel

Briefly, 30 g of MA was weighed and dissolved in 20 mL of ultrapure water at 4 °C with constant stirring until completely dissolved. After that, an additional 30 mL of cold water was added, and 20 g of P407 was gradually dispersed until completely dissolved. The cold water was added gradually, resulting in a total volume of 100 mL of solution. The final MA concentration was 30%. Moreover, a blank formulation (P407-gel) was prepared following the same process but without adding the drug.

### 4.3. MA-Gel Gelation Time Test

Next, 10 mL of MA-gel was added to a clear glass vial with a magnetic stir bar and placed in a 4 °C water bath. The solution was gradually heated under constant stirring at 400 rpm, and a thermometer was used to monitor the temperature. Gelation time was measured in triplicate once the magnetic stir bar stopped moving due to gelation of the gel, and the temperature data provided by the thermometer were recorded.

### 4.4. Physicochemical Characterization of MA-Gel

#### 4.4.1. Morphological Analysis

To analyze the morphology of the formulation, 0.5 g of the MA-gel was dried in a Petri dish for 4 days and then placed in a vacuum desiccator until it was completely dehydrated. Once dried, approximately 0.1 g of the dried MA-gel was coated with gold as a conductive agent. The internal structure of the MA-gel was examined by scanning electron microscopy (SEM) using a JEOL J-7100FE microscope (Peabody, MA, USA).

#### 4.4.2. pH Values

Due to P407’s thermoreversibility, the MA-gel was stored at 4 °C and 25 °C. The formulation’s pH values were measured using a Crison 501 digital pH/mV meter (Crison Instruments, Barcelona, Spain) for 90 days (days 1, 30, 60, and 90) at both temperatures. Values are expressed as the six replicates’ mean ± standard deviation (SD).

#### 4.4.3. Swelling and Degradation Test

**Figure 17:**
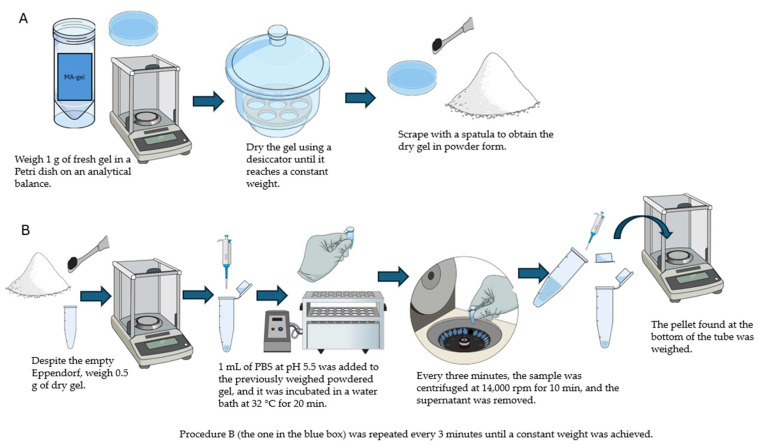
Procedure for performing the swelling test.

These tests were carried out employing a gravimetric method to determine the swelling ratio (SR) and assess the degradation, expressed as percent weight loss (WL). A 1 g sample of fresh MA-gel was weighed and then dried in a desiccator. Once the MA-gel was dry, 0.5 g was weighed into an Eppendorf tube and 1 mL of PBS (pH 5.5) was added at 32 °C for 20 min. The sample was centrifuged at 14,000 rpm for 10 min. The supernatant was removed, and 1 mL of PBS (pH 5.5) was added again. The procedure was repeated every 3 min until a constant weight was achieved (Figure 17).

**Figure 18:**
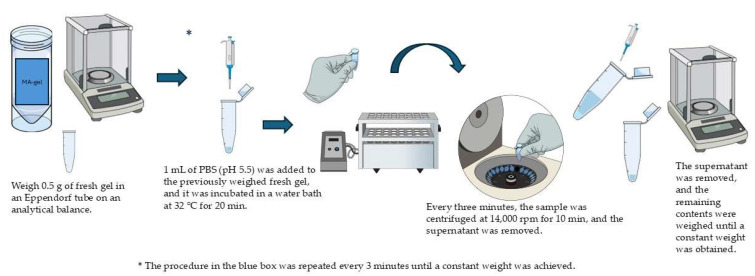
Procedure for performing the degradation test.

For the degradation test, the same procedure was followed as for the swelling test, with the difference that fresh gel was used (Figure 18).

The *SR* was calculated using the following equation and expressed using kinetic modeling:

(1) $$SR=\frac{Ws\;-\;Wd}{Wd}$$

*Ws* is the weight of the swollen MA-gel at 3 min intervals for 20 min, and *Wd* is the weight of the dry gel.

The percentage of WL was calculated using the equation and expressed through kinetic modeling.

(2) $$WL\%=\frac{Wi-Wd}{Wi}\;\times \;100$$

where *Wi* is the initial weight of the MA-gel and *Wd* is the weight of the gel at 3 min intervals for 20 min.

#### 4.4.4. Porosity Study

The percentage porosity (P) was calculated using a solvent displacement method. It consisted of immersing the previously dried MA-gel in absolute ethanol for 2 to 3 min and weighing it after removing excess ethanol from the surface. The percentage porosity was calculated using the following equation:

(3) $$P\%=\frac{W2\;-\;W1}{p\;\times \;V}\;\times \;100$$

where *W*1 represents the weight of the dry MA-gel, *W*2 represents the weight of the MA-gel after immersion in ethanol, *p* is the density of absolute ethanol, and *V* is the volume of the gel.

### 4.5. Rheological Measurements

Rheological characterization was performed using a Thermo Scientific Haake Rheostress 1 rotational rheometer (Thermo Fisher Scientific, Karlsruhe, Germany) equipped with a cone–plate system (Haake C60/2° Ti, diameter 60 mm, angle 2°, gap 0.105 mm). The temperature was controlled using a Haake Phoenix II + Haake C25P system. Data acquisition and analysis were conducted using Haake RheoWin^®^ Job Manager v.4.0 and RheoWin^®^ Data Manager v.4.0 software.

#### 4.5.1. Rotational Test

Viscosity (η) and flow (τ) curves were obtained at 4 °C and 32 °C using the following shear rate profile: ramp-up from 0 to 50 s^−1^ over 3 min, constant shear rate at 50 s^−1^ for 1 min, and ramp-down from 50 to 0 s^−1^ over 3 min.

Each measurement was duplicated. The flow data were fitted to the following rheological models: Newtonian, Bingham, Ostwald-De Waele, Cross, Casson, and Herschel–Bulkley. Viscosity values were extracted from the plateau region at 50 s^−1^.

#### 4.5.2. Oscillatory Test

Amplitude (stress) sweep tests were initially conducted at 1 Hz over a stress range of 0.1–200 Pa to identify the linear viscoelastic region (LVR) and assess the MA-gel strength through the critical stress value at 25 and 32 °C. A constant shear stress of 10 Pa within the LVR was selected for frequency sweep tests conducted from 0.01 to 10 Hz at 4 °C and 32 °C. The viscoelastic parameters measured included storage modulus (G′), loss modulus (G″), and complex viscosity (η*). A temperature sweep test was conducted from 10 to 40 °C at 1 Hz and 10 Pa.

### 4.6. Extensibility Test

To perform the MA-gel’s extensibility test, 0.5 g was placed inside a 1 cm diameter circle. Weights (5, 10, 20, 50, and 100 g) were added and left to stand for 2–3 min each at room temperature. The diameters (cm^2^) of the extended circles were measured and recorded for later graphical representation and adjustment to the best mathematical model.

### 4.7. Thermal Characterization and Compatibility Studies of MA-Gel by Differential Scanning Calorimetry (DSC)

DSC was used to evaluate possible interactions between MA and P407 using a Mettler-Toledo DSC-822e calorimeter. Experimental conditions: aluminum crucibles of 40 μL volume, atmosphere of dry nitrogen with 50 mL/min flow rate, heating rates of 10 °C/min. Differential scanning calorimetry was used to characterize both MA-gel and blank gel (P407-gel) at low temperatures using a Mettler-Toledo DSC5+/700 calorimeter. Experimental conditions: aluminum crucibles of 40 μL volume, atmosphere of dry nitrogen with 50 mL/min flow rate, heating rates of 5 °C/min. Both calorimeters were calibrated with indium of 99.99% purity (m.p.: 156.8 °C, ΔH: 28.59 J/g).

### 4.8. In Vitro Release Studies

The release of MA from the developed gel was evaluated using Franz vertical diffusion cells (FDC 400; Crown Glass, Somerville, NJ, USA) and hydrophilic polypropylene membranes (GH Polypro, Life Sciences). Water at 32 ± 0.5 °C was used as the receptor medium under immersion conditions, maintaining constant stirring at 600 rpm. The diffusion area was 0.64 cm^2^. A 0.3 g sample of MA-gel or 300 μL of MA solution was placed in the donor compartment. At the end of the experiment (after 400 min), the presence of SbV was assessed and quantified using inductively coupled plasma optical emission spectrometry (ICP-OES, PerkinElmer Elan 6000, Waltham, MA, USA). For this purpose, the samples were pretreated with HNO_3_ and H_2_O_2_ in a microwave digester at 220 °C for 72 h. Results were reported as the mean ± SD of three replicates.

### 4.9. Ex Vivo Permeation Studies

Ex vivo permeation tests were performed using healthy human skin. The Department of Plastic Surgery at Barcelona-SCIAS Hospital (Barcelona, Spain) provided human skin from the abdominal region of a healthy woman, following an experimental protocol (reference number: protocol N°002; dated 17 January 2020) approved by the Bioethics Committee of the same hospital. Human skin was sectioned using a dermatome (GA630 dermatome, Aesculap, Tuttlingen, Germany), resulting in 400 μm thick sections. The integrity of the skin samples was assessed in triplicate by measuring transepidermal water loss (TEWL) values using a DermaLab^®^ module (Cortex Technology, Hadsund, Denmark). An amount of 0.3 g of MA-gel was placed in the donor compartment using vertical Franz diffusion cells. Human skin was used as the test membrane, and water was used as the receptor medium. After 27 h of testing, the amount of SbV was calculated using ICP-OES in three replicates (*n* = 3).

To determine the amount of drug retained in the skin, the skin membranes were removed from the Franz cells, cleaned with gauze soaked in 0.05% sodium dodecyl sulfate solution, and gently washed three times with distilled water. The permeation areas were then excised and weighed precisely. Finally, the retained SbV was extracted in an ultrasonic bath using needle punctures and water for 30 min. ICP-OES was used to analyze the resulting solutions for quantification.

### 4.10. Microbiological Quality Control

The microbiological quality of MA-gel was evaluated using a 1:10 dilution of the MA-gel in peptone water, resulting in concentrations of 10^−1^, 10^−2^, and 10^−3^. Two 100 μL aliquots of each dilution was plated onto plates containing different culture media (*n* = 2).

The culture media used in this study included Sabouraud dextrose agar (SDA, for fungal and yeast growth), SMA (standard agar for mesophilic aerobic growth), and VRBA (red-violet-crystal neutral bile agar, for Gram-negative growth). Blood agar plates were simultaneously incubated in various concentrations to monitor the development of *Staphylococcus aureus* and *Pseudomonas aeruginosa*. All plates were incubated for 24 to 48 h at 37 ± 0.5 °C in a Thermo Scientific Heratherm (Waltham, MA, USA) incubator, while the SDA plates were incubated for 5 days at 25 °C (room temperature).

On the day of preparation, microbiological analysis was performed to identify mesophilic aerobic microorganisms, total coliforms, molds, and yeasts. After incubation, the plates were examined to observe colony development and determine the colony count per milliliter or gram of product. The growth of *Staphylococcus aureus* and *Pseudomonas aeruginosa* must be avoided.

### 4.11. In Vivo Tolerance Study

Biomechanical properties of the skin were evaluated in ten female volunteers with healthy skin between the ages of 25 and 35, who received topical treatment with the drug-free gel (P407 gel). The study was approved by the University of Barcelona Ethics Committee (reference number: IRB00003099; date: 30 January 2019) following the guidelines of the Declaration of Helsinki. All volunteers signed a written informed consent. No creams, lotions, or cosmetics were allowed on the test areas (forearms) for two days before the study. The volunteers remained in the testing room at 20 °C to prevent sweating for at least 30 min before the measurements. Transepithelial water loss (TEWL) measurements were performed using a Tewameter^®^ TM 300 (Courage-Khazaka electronic GmbH, Cologne, Germany). Stratum corneum hydration (SCH) was analyzed using a Corneometer^®^ CM 825 (Courage-Khazaka electronic GmbH). Measurements of TEWL and SCH were performed before the topical treatment to establish baseline readings and 15 min, 30 min, 1 h, and 3 h after applying 0.5 g of P407 gel on the flexor side of the left forearm.

### 4.12. In Vitro Cytotoxicity Assay

The cytotoxicity of MA solution, MA-gel, and P407 gel was evaluated using two cell lines, which included RAW 264.7 macrophages and HaCaT human skin keratinocytes. Firstly, 5 × 10^−1^ cells/mL of each cell line were cultured in 96-well plates (Costar 3595). Stepwise dilutions of the MA solution, MA-gel, and drug-free P407 gel were added to RPMI-1640 medium supplemented with heat-inactivated fetal bovine serum. Moreover, 1% penicillin (100 U/mL) and streptomycin (100 mg/mL) were added to prevent bacterial contamination. After 24 h of incubation at 37 °C with 5% CO_2_, WST-1 reagent (Roche Diagnostics GmbH, Mannheim, Germany) was added to each well, and the plate was maintained under the same conditions for an additional 4 h. The absorbance of the samples was recorded at 450 nm using a spectrophotometer (Multiskan EX, Thermo Electron Corporation, Shanghai, China). The concentration limiting cell viability to 50% (CC50) was initialized via linear regression analysis, and experiments were performed in triplicate.

### 4.13. In Vitro Antileishmanial Activity Against Promastigotes

The activity of MA-solution, MA-gel, and drug-free P407 gel against *Leishmania infantum* (MHOM/ES/2014/BCN-855) promastigotes was investigated using microtiter plates (Costar 3595). For this purpose, 100 µL of these samples were added to Schneider medium supplemented with 20% fetal bovine serum, 25 µg/mL gentamicin solution (Sigma, St. Louis, MO, USA), and 1% penicillin (100 U/mL)–streptomycin (100 mg/mL) solution (Sigma, St. Louis, MO, USA). Serial two-fold dilutions were prepared in the next 11 wells of each row. Subsequently, 100 µL of 10^6^ promastigotes/mL at their logarithmic growth stage (7 days) were incorporated into each well and incubated at 26 °C for 48 h. Briefly, samples were lysed, and growth was measured using acid phosphatase at an optical density of 406 nm using a spectrophotometer (Multiskan EX, Thermo Electron Corporation) (*n* = 3). The concentration that inhibited 50% of parasite growth (IC50) was determined by least squares linear regression analysis of parasite growth versus the logarithm of drug concentration with a 95% confidence interval.

### 4.14. In Vitro Antileishmanial Activity Against Intracellular Amastigotes

RAW 264.7 cells were grown at a concentration of 5 × 10^−1^ cells/mL in a LabTek eight-chamber slide system (Nunc^®^). After 24 h, Leishmania infantum promastigotes in the late stationary phase, obtained from a 6–7 day culture in RPMI-1640 complete medium supplemented with 10% heat-inactivated fetal bovine serum and 1% penicillin (100 U/mL)–streptomycin (100 µg/mL), were added at a concentration of 5 × 10^5^ cells/mL and incubated for 24 h at 37 °C in a 5% CO_2_ atmosphere. Free promastigotes were removed by washing with PBS. RPMI-1640 complete medium containing serial dilutions of the gel was added to each well and incubated at 37 °C in a 5% CO_2_ atmosphere for 48 h. Infected cells were washed, and slides were stained with Giemsa. Drug activity was assessed by counting the number of infected cells in triplicate samples of 300 macrophages.

### 4.15. Computational Studies: Molecular Docking Simulations

#### 4.15.1. Pharmacological Target Preparation

Given the chemical structure of MA (Figure 14A), exploring alternative pharmacological targets beyond the commonly proposed mechanism of action (i.e., the reduction of Sb(V) to Sb(III)) is of significant interest for new drug development and repurposing strategies [50,51,52]. In this study, we selected three relevant pharmacological targets: iron superoxide dismutase A (FeSODA), trypanothione reductase (TR), and CYP51, all of which have been implicated in the activity of various anti-leishmanial agents and have functional groups that can work as hydrogen bond acceptors. For docking validations, some reported ligands were employed. Examples include ZINC000253403245, C9 (4-(((5-((4-Fluorophenethyl)carbamoyl)furan-2-yl)methyl)(4-fluorophenyl)carbamoyl)-1-methyl-1-(3-phenylpropyl)piperazin-1-ium Iodide), and fluconazole, which are potential inhibitors of each protein, respectively, and were also employed to contrast the binding mode of MA with those of the cognate inhibitors. For FeSODA, due to the absence of a crystal structure in the RCSB Protein Data Bank (PDB), we utilized a homology model (ID: A4HTI0) from the Swiss Model repository (https://swissmodel.expasy.org/repository). This model was built based on the FeSODA template from Leishmania major (PDB ID: 4F2N), with a sequence identity of 92.61%. For TR and CYP51 from Leishmania infantum, the crystal structures (PDB IDs: 2JK6 and 3L4D, respectively) were obtained from the RCSB-PDB and prepared by removing non-essential co-crystallized molecules (e.g., water, salts) adding polar hydrogens and partial charges, with no further structural processing. Moreover, to analyze the potential of the triblock copolymer composed of poly(ethylene oxide) (PEO) and poly(propylene oxide) (PPO) in a PEO-PPO-PEO configuration, known as P407, as a drug delivery system, we also performed docking studies between this system (with a monomer) and MA to characterize the chemical interactions governing the molecular recognition of this pharmaceutical carrier toward MA.

#### 4.15.2. Ligand Preparation and Molecular Docking Studies

The 2D structure of MA, a monomer of P407, and the potential inhibitors of FeSODa, TR and CYP51 (ZINC000253403245, C9 (4-(((5-((4-Fluorophenethyl)carbamoyl)furan-2-yl)methyl)(4-fluorophenyl)carbamoyl)-1-methyl-1-(3-phenylpropyl)piperazin-1-ium Iodide), and fluconazole, respectively), were drawn using ChemSketch Freeware (https://www.acdlabs.com/resources/free-chemistry-software-apps/chemsketch-freeware/ accessed on 20 May of 2025). A preliminary 3D optimization was performed, and the structures were saved in .mol format. Then, the files were converted to a Z-matrix format using GaussView 5.0, followed by geometry and energy optimization, including the correct protonation state assignment, using the B3LYP/3-21G (for AM) and AM1 (for the remaining ligands) methods within the Gaussian 09 suite. The optimized outputs were then converted into .pdb files for molecular docking. Docking simulations were conducted using AutoDock 4.2.6. Prior to docking simulations, polar hydrogens were added to all protein hetero-atoms capable of forming polar interactions, and Kollman charges were assigned across the protein. For the ligands, Gasteiger charges were used. Affinity maps were generated using a focused docking approach within a grid box of 60 Å^3^ in each dimension (X, Y, Z) with a grid center for each pharmacological target positioned over the residues that formed the catalytic center, with a grid spacing of 0.374 Å^3^. The Lamarckian genetic algorithm was employed for the docking search using an initial population of 100 individuals and a maximum of 1 × 10^7^ energy evaluations, retaining one elite individual per generation. Protein–ligand interactions and 3D visualizations were analyzed using PyMOL Molecular Graphics System, Version 3.0 Schrödinger, LLC (Figure 14B).

## Figures and Tables

**Table 1:** Leishmanicidal activity of MA-solution and MA-gel against promastigotes and amastigotes of *L. infantum.*

IC50 (µg/mL)					
Strain	Compounds	Promastigote	Amastigote	CC50 * Raw 264.7	SI **
*L. infantum*	MA-solution	6.96 ± 15.98	112.09 ± 20.73	253.03 ± 55.37	2.25
	MA-gel	3.56 ± 0.22	23.11 ± 12.43	196.05 ± 30.89	8.48
	P407	Without effect	Without effect		

* Cytotoxic concentration 50; ** selectivity index.

**Table 2:** Interacting residues from the MA and inhibitor/target complexes analyzed by molecular docking simulations. ^∆^ depicts a residue forming an h-bond.

Target	Ligand	Binding Free Energy Values (kcal/mol)	Residues Reached by Ligands
FeSODA	MA	−3.9	H62, S154^∆^, W193, E194^∆^, H195^∆^, Y198
	ZINC000253403245	−6.46	L57, H62^∆^, Q54, E194, H195, Y198, K199
Trypanothione reductase	MA	−5.03	K61^∆^, L334, P336, A363, C364, E436^∆^, Q439^∆^, F367, FAD^∆^
	C9	−7.9	C57, V58, K61^∆^, P335, P336, V337, N340, T357, D358, V362, P435, Q439, FAD
CYP51	MA	−4.58	Y115, P209, A290, T294, L355, M357^∆^, HEME
	Fluconazole	−7.45	Y102, F109, Y115, L125, A286, A290, T294, L355, HEME

## Data Availability

The data presented in this study are available in this article.

## Supplementary Materials

The following supporting information can be downloaded at: https://www.mdpi.com/article/10.3390/gels11080601/s1, Figure S1: DSC thermogram of pure meglumine antimoniate; Figure S2: DSC thermogram of pure pluronic acid; Figure S3: DSC thermogram of the mixture of the components of the formulation in the original proportion.

## References

[1] Cecílio P., Cordeiro-da-Silva A., Oliveira F. (2022). Sand flies: Basic information on the vectors of leishmaniasis and their interactions with Leishmania parasites. Commun. Biol..

[2] Mann S., Frasca K., Scherrer S., Henao-Martínez A.F., Newman S., Ramanan P., Suarez J.A. (2021). A Review of Leishmaniasis: Current Knowledge and Future Directions. Curr. Trop. Med. Rep..

[3] Marcondes M., Day M.J. (2019). Current status and management of canine leishmaniasis in Latin America. Res. Vet. Sci..

[4] Abadías-Granado I., Diago A., Cerro P.A., Palma-Ruiz A.M., Gilaberte Y. (2021). Cutaneous and Mucocutaneous Leishmaniasis. Actas Dermo-Sifiliogr..

[5] Bezemer J.M., van der Ende J., Limpens J., de Vries H.J.C., Schallig H. (2021). Safety and efficacy of allylamines in the treatment of cutaneous and mucocutaneous leishmaniasis: A systematic review. PLoS ONE.

[6] Meireles C.B., Maia L.C., Soares G.C., Teodoro I.P.P., Gadelha M., da Silva C.G.L., de Lima M.A.P. (2017). Atypical presentations of cutaneous leishmaniasis: A systematic review. Acta Trop..

[7] Remadi L., Haouas N., Chaara D., Slama D., Chargui N., Dabghi R., Jbeniani H., Mezhoud H., Babba H. (2016). Clinical Presentation of Cutaneous Leishmaniasis caused by Leishmania major. Dermatology.

[8] Mokni M. (2019). Leishmanioses cutanées. Ann. Dermatol. Venereol..

[9] Scott P., Novais F.O. (2016). Cutaneous leishmaniasis: Immune responses in protection and pathogenesis. Nat. Rev. Immunol..

[10] Elmahallawy E.K., Alkhaldi A.A.M., Saleh A.A. (2021). Host immune response against leishmaniasis and parasite persistence strategies: A review and assessment of recent research. Biomed. Pharmacother. Biomed. Pharmacother..

[11] Carvalho S.H., Frézard F., Pereira N.P., Moura A.S., Ramos L., Carvalho G.B., Rocha M.O.C. (2019). American tegumentary leishmaniasis in Brazil: A critical review of the current therapeutic approach with systemic meglumine antimoniate and short-term possibilities for an alternative treatment. Trop. Med. Int. Health.

[12] Fernandes H.J., da Silva R.E., Ramalho D.B., Aguiar M.G., Silveira J.N., Cota G. (2020). Safety profile of meglumine antimoniate intralesional infiltration for cutaneous leishmaniasis. Expert Rev. Anti-Infect. Ther..

[13] Zarrintaj P., Ramsey J.D., Samadi A., Atoufi Z., Yazdi M.K., Ganjali M.R., Amirabad L.M., Zangene E., Farokhi M., Formela K. (2020). Poloxamer: A versatile tri-block copolymer for biomedical applications. Acta Biomater..

[14] Martins P.S., Ochoa R., Pimenta A.M.C., Ferreira L.A.M., Melo A.L., da Silva J.B.B., Sinisterra R.D., Demicheli C., Frézard F. (2006). Mode of action of β-cyclodextrin as an absorption enhancer of the water-soluble drug meglumine antimoniate. Int. J. Pharm..

[15] Jaser M.A., El-Yazigi A., Croft S.L. (1995). Pharmacokinetics of antimony in patients treated with sodium stibogluconate for cutaneous leishmaniasis. Pharm. Res..

[16] Cortez-Maya S., Moreno-Herrera A., Palos I., Rivera G. (2020). Old Antiprotozoal Drugs: Are They Still Viable Options for Parasitic Infections or New Options for Other Diseases?. Curr. Med. Chem..

[17] de Vries H.J.C., Schallig H.D. (2022). Cutaneous Leishmaniasis: A 2022 Updated Narrative Review into Diagnosis and Management Developments. Am. J. Clin. Dermatol..

[18] Singh Malik D., Mital N., Kaur G. (2016). Topical drug delivery systems: A patent review. Expert Opin. Ther. Pat..

[19] Berenguer D., Sosa L., Alcover M., Sessa M., Halbaut L., Guillén C., Fisa R., Calpena-Campmany A.C., Riera C. (2019). Development and Characterization of a Semi-Solid Dosage Form of Meglumine Antimoniate for Topical Treatment of Cutaneous Leishmaniasis. Pharmaceutics.

[20] Aragão Horoiwa T., Cortez M., Sauter I.P., Migotto A., Bandeira C.L., Cerize N.N.P., de Oliveira A.M. (2020). Sugar-based colloidal nanocarriers for topical meglumine antimoniate application to cutaneous leishmaniasis treatment: Ex vivo cutaneous retention and in vivo evaluation. Eur. J. Pharm. Sci. Off. J. Eur. Fed. Pharm. Sci..

[21] Pereira M.B., Sydor B.G., Memare K.G., Verzignassi Silveira T.G., Alessi Aristides S.M., Dalmarco E.M., Vieira Teixeira J.J., Campana Lonardoni M.V., Demarchi I.G. (2021). In vivo efficacy of meglumine antimoniate-loaded nanoparticles for cutaneous leishmaniasis: A systematic review. Nanomedicine.

[22] Kumari P., Kant V., Chandratre G.A., Ahuja M. (2024). Formulation and Evaluation of Pluronic F-127 Thermoresponsive Nanogels Containing Juglone for In vivo Wound Healing Potential. BioNanoScience.

[23] Suman K., Sourav S., Joshi Y.M. (2021). Rheological signatures of gel–glass transition and a revised phase diagram of an aqueous triblock copolymer solution of Pluronic F127. Phys. Fluids.

[24] Sosa L., Espinoza L.C., Silva-Abreu M., Jaramillo-Fierro X., Berenguer D., Riera C., Rincón M., Calpena A.C. (2025). In Vitro Efficacy and Toxicity Assessment of an Amphotericin B Gel for the Treatment of Cutaneous Leishmaniasis. Pharmaceuticals.

[25] Sosa L., Calpena A.C., Silva-Abreu M., Espinoza L.C., Rincón M., Bozal N., Domenech O., Rodríguez-Lagunas M.J., Clares B. (2019). Thermoreversible Gel-Loaded Amphotericin B for the Treatment of Dermal and Vaginal Candidiasis. Pharmaceutics.

[26] Espinoza L.C., Guaya D., Calpena A.C., Perotti R.M., Halbaut L., Sosa L., Brito-Llera A., Mallandrich M. (2022). Comparative Study of Donepezil-Loaded Formulations for the Treatment of Alzheimer’s Disease by Nasal Administration. Gels.

[27] Foudazi R., Zowada R., Manas-Zloczower I., Feke D.L. (2023). Porous Hydrogels: Present Challenges and Future Opportunities. Langmuir ACS J. Surf. Colloids.

[28] Roberge C.L., Kingsley D.M., Cornely L.R., Spain C.J., Fortin A.G., Corr D.T. (2023). Viscoelastic Properties of Bioprinted Alginate Microbeads Compared to Their Bulk Hydrogel Analogs. J. Biomech. Eng..

[29] Silva-Abreu M., Sosa L., Espinoza L.C., Fábrega M.-J., Rodríguez-Lagunas M.J., Mallandrich M., Calpena A.C., Garduño-Ramírez M.L., Rincón M. (2023). Efficacy of Apremilast Gels in Mouse Model of Imiquimod-Induced Psoriasis Skin Inflammation. Pharmaceutics.

[30] Lupu A., Gradinaru L.M., Rusu D., Bercea M. (2023). Self-Healing of Pluronic® F127 Hydrogels in the Presence of Various Polysaccharides. Gels.

[31] White J.M., Garza A., Griebler J.J., Bates F.S., Calabrese M.A. (2023). Engineering the Structure and Rheological Properties of P407 Hydrogels via Reverse Poloxamer Addition. Langmuir ACS J. Surf. Colloids.

[32] Grassi G., Crevatin A., Farra R., Guarnieri G., Pascotto A., Rehimers B., Lapasin R., Grassi M. (2006). Rheological properties of aqueous Pluronic–alginate systems containing liposomes. J. Colloid Interface Sci..

[33] Balestrieri F., Magrì A.D., Magrì A.L., Marini D., Sacchini A. (1996). Application of differential scanning calorimetry to the study of drug-excipient compatibility. Thermochim. Acta.

[34] Mura P., Manderioli A., Bramanti G., Furlanetto S., Pinzauti S. (1995). Utilization of differential scanning calorimetry as a screening technique to determine the compatibility of ketoprofen with excipients. Int. J. Pharm..

[35] Gill P., Moghadam T.T., Ranjbar B. (2010). Differential scanning calorimetry techniques: Applications in biology and nanoscience. J. Biomol. Tech. JBT.

[36] Giron D. (1986). Applications of thermal analysis in the pharmaceutical industry. J. Pharm. Biomed. Anal..

[37] Dar M.J., Din F.U., Khan G.M. (2018). Sodium stibogluconate loaded nano-deformable liposomes for topical treatment of leishmaniasis: Macrophage as a target cell. Drug Deliv..

[38] Moosavian Kalat S.A., Khamesipour A., Bavarsad N., Fallah M., Khashayarmanesh Z., Feizi E., Neghabi K., Abbasi A., Jaafari M.R. (2014). Use of topical liposomes containing meglumine antimoniate (Glucantime) for the treatment of L. major lesion in BALB/c mice. Exp. Parasitol..

[39] Ricci E.J., Bentley M.V.L.B., Farah M., Bretas R.E.S., Marchetti J.M. (2002). Rheological characterization of Poloxamer 407 lidocaine hydrochloride gels. Eur. J. Pharm. Sci..

[40] Koffi A.A., Agnely F., Ponchel G., Grossiord J.L. (2006). Modulation of the rheological and mucoadhesive properties of thermosensitive poloxamer-based hydrogels intended for the rectal administration of quinine. Eur. J. Pharm. Sci..

[41] Choi H.-G., Lee M.-K., Kim M.-H., Kim C.-K. (1999). Effect of additives on the physicochemical properties of liquid suppository bases. Int. J. Pharm..

[42] Park Y.-J., Yong C.S., Kim H.-M., Rhee J.-D., Oh Y.-K., Kim C.-K., Choi H.-G. (2003). Effect of sodium chloride on the release, absorption and safety of diclofenac sodium delivered by poloxamer gel. Int. J. Pharm..

[43] Li X., Li A., Feng F., Jiang Q., Sun H., Chai Y., Yang R., Wang Z., Hou J., Li R. (2019). Effect of the hyaluronic acid-poloxamer hydrogel on skin-wound healing: In vitro and in vivo studies. Anim. Models Exp. Med..

[44] Cui N., Dai C.-Y., Mao X., Lv X., Gu Y., Lee E.-S., Jiang H.-B., Sun Y. (2022). Poloxamer-Based Scaffolds for Tissue Engineering Applications: A Review. Gels.

[45] Brugués A.P., Naveros B.C., Calpena Campmany A.C., Pastor P.H., Saladrigas R.F., Lizandra C.R. (2015). Developing cutaneous applications of paromomycin entrapped in stimuli-sensitive block copolymer nanogel dispersions. Nanomedicine.

[46] Bora K., Sarma M., Kanaujia S.P., Dubey V.K. (2024). Development of novel dual-target drugs against visceral leishmaniasis and combinational study with miltefosine. Free Radic. Biol. Med..

[47] Exertier C., Salerno A., Antonelli L., Fiorillo A., Ocello R., Seghetti F., Caciolla J., Uliassi E., Masetti M., Fiorentino E. (2024). Fragment Merging, Growing, and Linking Identify New Trypanothione Reductase Inhibitors for Leishmaniasis. J. Med. Chem..

[48] Hargrove T.Y., Wawrzak Z., Liu J., Nes W.D., Waterman M.R., Lepesheva G.I. (2011). Substrate preferences and catalytic parameters determined by structural characteristics of sterol 14alpha-demethylase (CYP51) from Leishmania infantum. J. Biol. Chem..

[49] Baiocco P., Colotti G., Franceschini S., Ilari A. (2009). Molecular Basis of Antimony Treatment in Leishmaniasis. J. Med. Chem..

[50] López-Arencibia A., Bethencourt-Estrella C.J., Berenguer D., Domínguez-de-Barros A., Alcover M.M., Sessa M., Halbaut L., Fisa R., Calpena-Campmany A.C., Córdoba-Lanús A.E. (2024). In Vivo Evaluation of Sepigel-Based Meglumine Antimoniate and Amphotericin B for Cutaneous Leishmaniasis Treatment. Pathogens.

[51] Guarimata J.D., Lavecchia M. (2024). In Silico Study of FDA-Approved Drugs on Leishmania infantum CYP51, a Drug Repositioning Approach in Visceral Leishmaniasis. Chem. Proc..

[52] Bora K., Sarma M., Kanaujia S.P., Dubey V.K. (2023). Dual-target drugs against Leishmania donovani for potential novel therapeutics. Sci. Rep..

