# Supplementary material for: Topical Meglumine Antimoniate Gel for Cutaneous Leishmaniasis: Formulation, Evaluation, and In Silico Insights
Source: Gels. 2025 Aug 1;11(8):601. doi: 10.3390/gels11080601 (PMC12385701; doi:10.3390/gels11080601)
Supplement: Supplementary file 1 [file gels-11-00601-s001.zip › gels-3743036-supplementary.pdf]

Supplementary Information

Characterization of the gels by Differential scanning calorimetry (DSC)

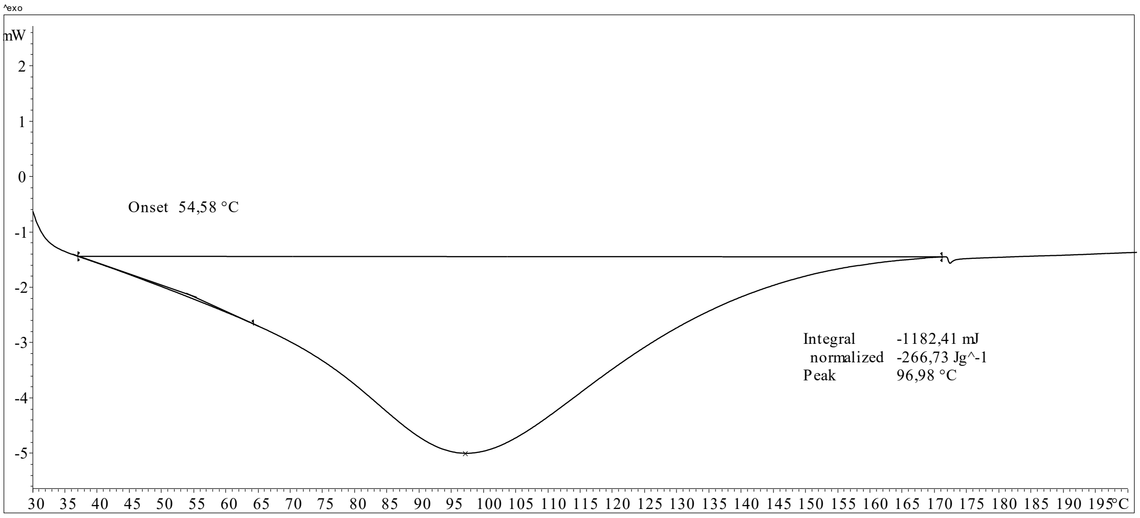

Figure S1: DSC thermogram of pure meglumine antimoniate.

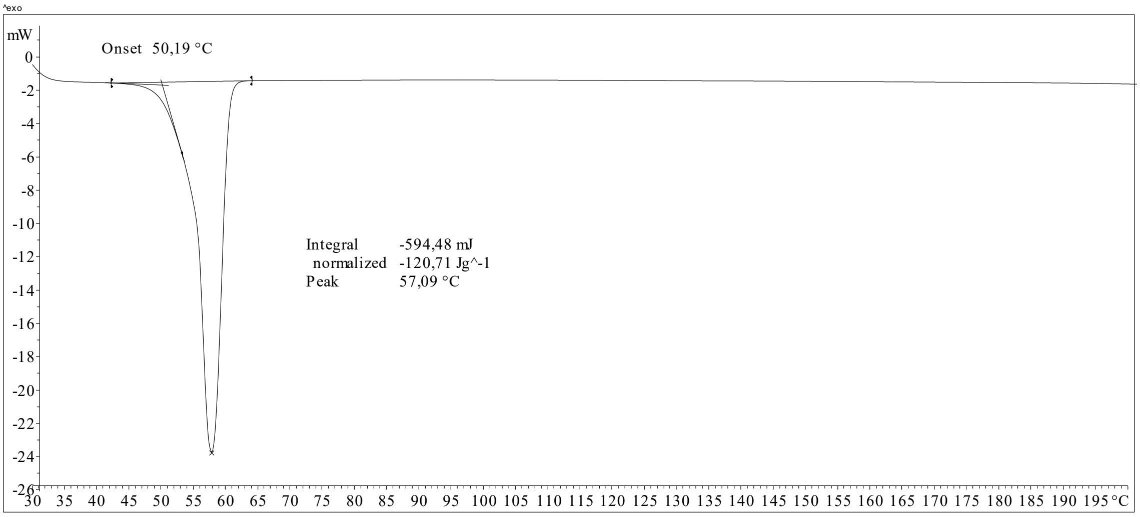

Figure S2: DSC thermogram of pure pluronic acid.

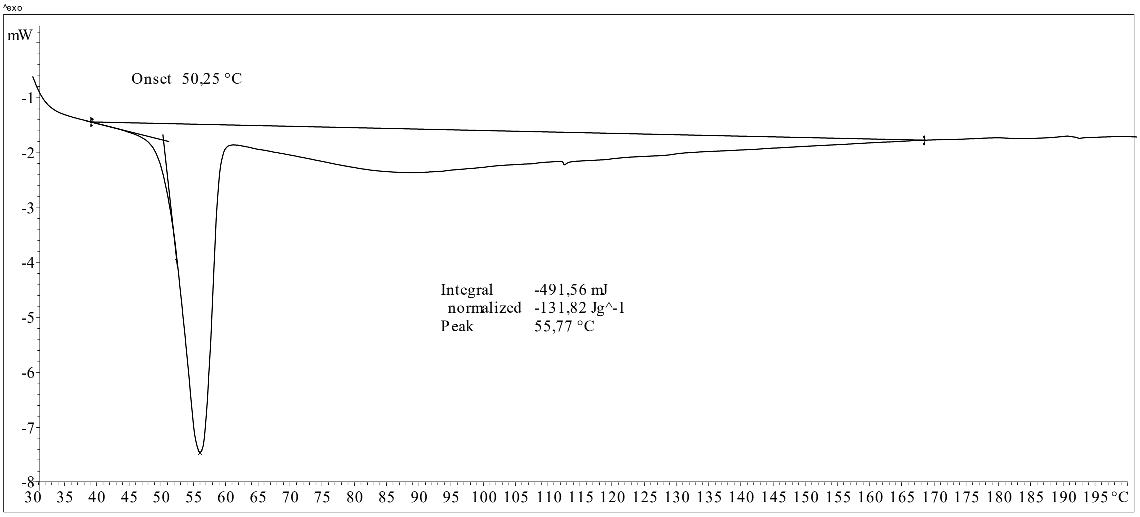

Figure S3: DSC thermogram of the mixture of the components of the formulation in the original proportion.
